# Lattice Defects and Exfoliation Efficiency of 6H-SiC via H_2_^+^ Implantation at Elevated Temperature

**DOI:** 10.3390/ma13245723

**Published:** 2020-12-15

**Authors:** Tao Wang, Zhen Yang, Bingsheng Li, Shuai Xu, Qing Liao, Fangfang Ge, Tongmin Zhang, Jun Li

**Affiliations:** 1 Institute of Fluid Physics, China Academy of Engineering Physics, Mianyang 621900, China; 2 Sino-French Institute of Nuclear Engineering and Technology, Sun Yat-Sen University, Zhuhai 519082, China; 3 State Key Laboratory for Environment-Friendly Energy Materials, Southwest University of Science and Technology, Mianyang 621010, China; xushuai@swust.edu.cn (S.X.); Liaoq@swust.edu.cn (Q.L.); 4 Ningbo Institute of Materials Technology and Engineering, Chinese Academy of Sciences, Ningbo 315201, China; gefangfang@nimte.ac.cn; 5 Institute of Modern Physics, Chinese Academy of Science, Lanzhou 730000, China; zhangtm@impcas.ac.cn (T.Z.); lijun@impcas.ac.cn (J.L.)

**Keywords:** 6H-SiC, H_2_^+^ implantation, exfoliation, microstructure

## Abstract

Silicon carbide (SiC) is an important material used in semiconductor industries and nuclear power plants. SiC wafer implanted with H ions can be cleaved inside the damaged layer after annealing, in order to facilitate the transfer of a thin SiC slice to a handling wafer. This process is known as “ion-cut” or “Smart-Cut”. It is worth investigating the exfoliation efficiency and residual lattice defects in H-implanted SiC before and after annealing. In the present paper, lattice damage in the 6H-SiC implanted by H_2_^+^ to a fluence of 5 × 10^16^ H_2_^+^/cm^2^ at 450 and 900 °C was investigated by a combination of Raman spectroscopy and transmission electron microscopy. Different levels of damage caused by dynamic annealing were observed by Raman spectroscopy and transmission electron microscopy in the as-implanted sample. Atomic force microscopy and scanning white-light interferometry were used to observe the sample surface morphology. Surface blisters and exfoliations were observed in the sample implanted at 450 °C and then annealed at 1100 °C for 15 min, whereas surface blisters and exfoliation occurred in the sample implanted at 900 °C without further thermal treatment. This finding can be attributed to the increase in the internal pressure of platelets during high temperature implantation. The exfoliation efficiency, location, and roughness after exfoliation were investigated and possible reasons were discussed. This work provides a basis for further understanding and improving the high-efficiency “ion-cut” technology.

## 1. Introduction

Silicon carbide (SiC) is regarded as one of the most important wide-band gap semiconductors due to its excellent physical, electronic, and optical performances, i.e., a high melting temperature, a high strength, a high thermal conductivity, a large breakdown voltage, and a high electron mobility [1,2]. Much effort has been made to develop the potential applications of SiC devices, such as Schottky barrier diodes in next-generation, large-scale integrated circuits. Although SiC has more than 200 polytypes, the hexagonal 4H- and 6H-SiC are particularly promising due to their advanced physical properties.

To reduce the high cost of SiC wafers and improve SiC-devices, SiC-on-insulator (SiCOI) structures have been proposed because of their excellent performance, such as the low-power dissipation to save energy and the high radiation resistance to use in space [3]. Similar to many other semiconductors, SiCOI structures can be fabricated by “smart-cut” technology, which was first reported by Bruel [4] in 1995, to achieve silicon layer transfer for the fabrication of silicon-on-insulator (SOI) materials. The “Smart-Cut” technology contains three main processes, initially hydrogen or helium ion implantation with a fluence of the order of 10^16^ to 10^17^ cm^−2^ at room temperature, then wafer bonding to another rigid substrate (handling wafer) before thermal annealing, and finally fracture to achieve thin layer transfer at elevated temperatures [5,6,7]. The initiation and propagation of micro-cracks in H-implanted SiC play a critical role in exfoliation of the wafer surface. The formation of micro-cracks depends on the growth of platelets. These platelets are composed of vacancy-hydrogen compounds. The formation of vacancy-hydrogen compounds is due to the interaction between implantation-induced vacancies and implanted hydrogen. Therefore, it is critical to investigate the formation and growth of platelets in SiC implanted with H ions under different experimental conditions, such as the implantation fluence, temperature, and annealing treatment. It is well known that the growth of micro-cracks inside the SiC wafer can induce surface blisters when the SiC wafer is not bonded to a substrate, and the same activation energy between blister formation and layer splitting is argued by Tong et al. [8]; therefore, it is a convenient way to evaluate the smart-cut threshold condition via observation of surface blisters and exfoliation. Our recent study involved 6H-SiC implanted by 134 keV H_2_^+^ at room temperature [9]. The maximum exfoliation efficiency was achieved for the sample implanted with a fluence of 1.5 × 10^16^ H_2_^+^/cm^2^ followed by 1100 °C annealing for 15 min. A further increase in implantation fluence was found to retard the exfoliation efficiency due to the negative effects of implantation-induced lattice damage on the growth of vacancy-hydrogen clusters, consistent with the report of Gregory et al. [10] that the threshold fluence for exfoliation in H-implanted 4H-SiC decreases with increasing implantation temperature (room temperature to 600 °C). Up to now, most of the published reports aimed at H-implanted SiC were concerned with implantation at a low temperature and then annealing at a high temperature [11,12,13,14,15,16,17]. The exfoliation effect in H-implanted SiC without the annealing treatment was, to our knowledge, not investigated. Many open questions are concerned with the nature of the H implantation-induced defects and their influence on micro-crack growth. In this paper, we studied the exfoliation efficiency of 6H-SiC implanted at 450 °C and subsequently annealed at 1100 °C for 15 min, compared with 6H-SiC implanted at 900 °C without annealing.

## 2. Experimental Process

**Figure 1:**
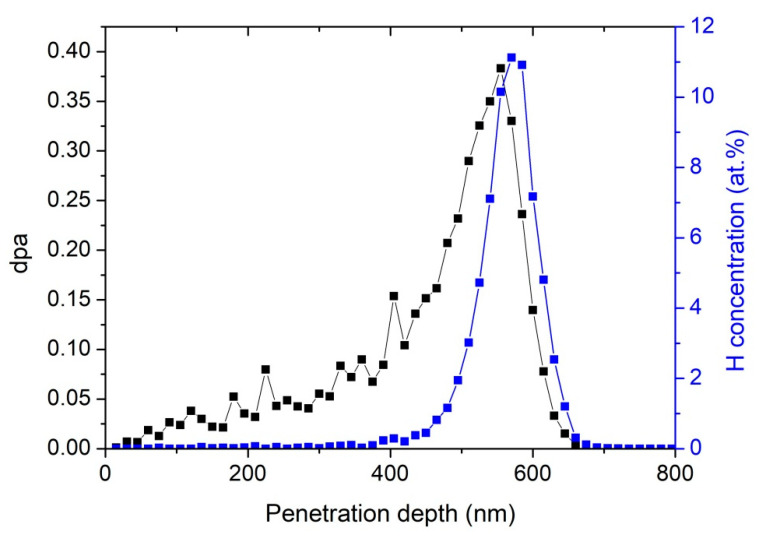
Depth distributions of the displacements per atom (dpa) and the projected range of 194 keV H_2_^+^-implanted 6H-SiC to a fluence of 5 × 10^16^ H_2_^+^/cm^2^ simulated using the SRIM-2013 code (density of 3.21 g/cm^3^ and displacement energies of C = 20 eV and Si = 35 eV).

For the experiments to study the exfoliation efficiency of SiC as a function of implantation temperature, bulk SiC samples, 6H polytype <0001>_Si_ orientation, purchased as research grade material from HF-Kejing Company, Heifei, China, were implanted with 194 keV H_2_^+^ to a fluence of 5 × 10^16^ H_2_^+^/cm^2^ at 450 and 900 °C. Hydrogen implantation experiments were performed on a 320 kV high-voltage platform equipped with ECR (Electron Cyclotron Resonance) ion sources in the Institute of Modern Physics, Chinese Academy of Sciences (CAS). The beam was rastered using an electrostatic scanner with fixed frequencies of 993 and 990 Hz in horizontal and vertical directions, respectively, to provide uniform ion fluence across the sample. The ion fluence was in-situ measured using a Faraday cup assembly in front of the sample. The beam flux was kept at 2.3 × 10^13^ ions/cm^2^ s. The implantation temperature was measured by a thermocouple, and the deviation of the implantation temperature was less than 1 °C. The wafers were tilted 7–8° from the direction of normal incidence during the implantation. According to the Stopping and Range of Ions in Matter (SRIM-2013) [18], the expected H peak concentration was approximately 11 at.% at 576 nm below the sample surface, as shown in Figure 1. To observe exfoliation on the surface of H-implanted 6H-SiC at 450 °C, thermal annealing at 1100 °C for 15 min in air atmosphere was performed.

Lattice damage before and after annealing was investigated by Raman spectroscopy and transmission electron microscopy (TEM) using a Tecnai G20 operated at 200 kV. Confocal Raman spectra were recorded at room temperature in a ***z***(***xx***)***z*** backscattering geometry using an HR-800 spectrometer from France. The 532 nm line of an argon ion laser was focused on a 1 × 1 μm^2^ spot and collected through a 50× objective lens. A 100 μm confocal pinhole diameter was used, and 600 lines/mm grating were performed. The acquisition time for each spectrum was 30 s for one accumulation. The spectra were measured ranging from 150 to 1800 cm^−1^. A double tilt goniometer stage was used, in order to tilt the TEM sample to satisfy different diffraction vectors. The lattice defects were detected by weak-beam dark-field (WBDF) with (***g***, 3***g***), ***g*** = 0002 and ***g*** = 2110 near ***z*** = 0110, where ***g*** is the diffraction vector and ***z*** is the zone axis. To study the depth distribution of implantation-induced defects, cross-sectional samples were prepared. The fabrication process of the cross-sectional transmission electron microscopy (XTEM) samples was described as follows. Initially, XTEM samples were prepared by mechanical thinning up to approximately 30 µm in thickness, followed by ion milling with Ar ions in two steps. In the first step, the ion milling energy was 5 kV with a glancing angle of ±5° until optically controlled perforation occurred in the middle of the XTEM sample. In the second step, ion milling energy decreased to 2 kV with a glancing angle of ± 3° for 1 h to minimize radiation damage induced by the Ar ions [14,15]. The surface morphology was measured by scanning white-light interferometry (SWLI), and surface roughness after exfoliation was measured by atomic force microscopy (AFM).

## 3. Results

**Figure 2:**
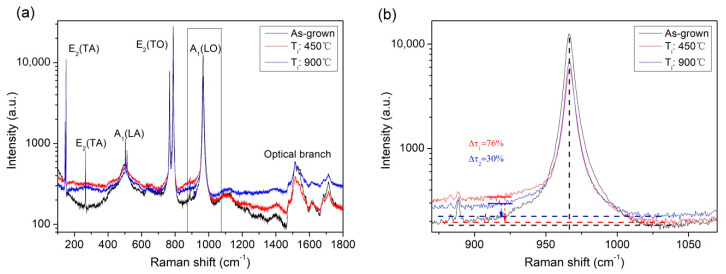
Raman spectra of (**a**) 6H-SiC implanted by H_2_^+^-ion at 450 °C and 900 °C showing first-order (E_2_(TO) and A_1_(LO)) and second-order (E_2_(TA), E_1_(TA), A_1_(LA) and optical branch) peaks, compared with the as-grown 6H-SiC, (**b**) enlarged A_1_(LO) peak shown in figure (**a**), where the left tail of the peak lifted after ion implantation. The intensity of the asymmetry decreased with increasing implantation temperature.

Figure 2 presents the Raman spectra of the 194 keV H_2_^+^-implanted 6H-SiC to a fluence of 5 × 10^16^ H_2_/cm^2^ at 450 and 900 °C. In the as-grown 6H-SiC, some Raman scattering peaks were clearly visible. Nakashima and Harima [19] investigated the Raman scattering of SiC crystals, and they found that the Raman-active models of the wurtzite structure were the A_1_, E_1_, and E_2_ modes. In addition, the A_1_ and E_2_ phonon modes can be split into transverse acoustic (TA) and optical (TO), as well as longitudinal acoustic (LA) and optical (LO) modes. The first-order Raman bands assigned to E_2_(TO) at 767 and 789 cm^−1^ and A_1_(LO) at 967 cm^−1^ were observed [20]. Besides the first-order Raman bands, second-order Raman bands attributed to E_2_(TA) at 146 and 150 cm^−1^, E_2_(TA) at 266 cm^−1^, and A_1_(LA) at 504 and 513 cm^−1^ [20]. The Raman active located in the 1500–1750 cm^−1^ region can be attributed to optical branching [20]. The strong intensity of the second-order Raman bands indicates the good quality of the wafer. After H_2_^+^ ion implantation, the intensities of the first-order Raman bands and the second-order Raman bands decreased. This finding can be assigned to the increase in the optical absorption coefficient of 6H-SiC after H implantation [21,22]. It is a simple method to evaluate the lattice disorder by means of the change in Raman scattering intensity. The Raman scattering of A_1_(LO) is enlarged and shown in Figure 2b. Compared with the H_2_^+^-implanted 6H-SiC at 900 °C, in H_2_^+^-implanted 6H-SiC at 450 °C, the Raman scattering decreased more significantly. Moreover, the asymmetric broadening of the A_1_(LO) peak can be observed. In detail, the left tail of the A_1_(LO) peak lifted after H_2_^+^ ion implantation. The intensity of the asymmetry can be expressed as Δτ = (*I*_left_ − *I*_right_)/*I*_right_, where *I* is the intensity of the Raman scattering baseline; Δτ equaled 76% and 30% at the 450 °C and 900 °C implantation, respectively. The asymmetric broadening of the A_1_(LO) peak can be accounted for by a “spatial correlation” model where implantation-induced defects can induce *q*-vector relaxation [23,24]. The more lattice defects in the wafer, the stronger the asymmetric broadening that can be formed [25]. Therefore, it is reasonable to assume that the number density of lattice defects formed at 450 °C implantation is larger than that of the sample implanted at 900 °C. It is easily explained that the dynamic annealing increases with increasing implantation temperature.

**Figure 3:**
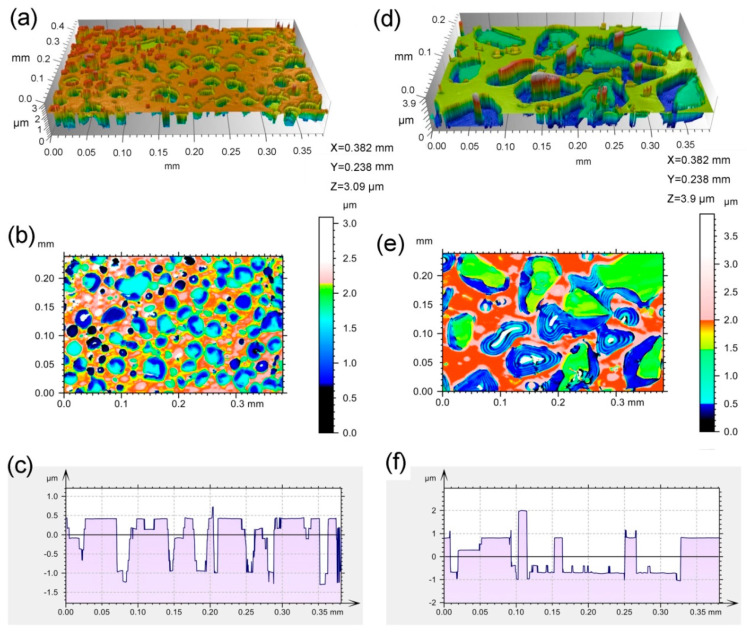
(**a**,**d**) show surface morphology observed in the three-dimensional profile, (**b**,**d**) show the height distribution in a two-dimensional profile, and (**c**,**f**) show the contour curve obtained from figures (**b**,**d**), respectively. (**a**–**c**) are the 194 keV H_2_^+^-implanted 6H-SiC at 450 °C followed by 1100 °C annealing for 15 min. (**d**–**f**) are the 194 keV H_2_^+^-implanted 6H-SiC at 900 °C.

Figure 3 shows the surface morphology of the 194 keV H_2_^+^-implanted SiC by means of the scanning white-light interferometry method. In the 6H-SiC implanted with H_2_^+^ ions at 450 °C after 1100 °C annealing for 15 min, surface exfoliation was clearly observed in Figure 3a,d. Surface blisters were observed in the two-dimensional profile shown in Figure 3b,f. Exfoliation depth and size were analyzed by a contour curve, as shown in Figure 3c,g. It can be seen that most of the exfoliation depth is near 1.0 μm for the H_2_^+^-implanted 6H-SiC at 450 °C followed by 1100 °C annealing, while it is near 0.8 μm for the H_2_^+^-implanted 6H-SiC at 900 °C It should be noted that the exfoliation depth observed by scanning white light interferometry is not exact due to lattice swelling induced by surface blisters. Moreover, the size of the exfoliation zone is in the range of 10 to 40 μm for the H_2_^+^-implanted 6H-SiC at 450 °C followed by 1100 °C annealing, while it is in the range of 20 to 100 μm for the 6H-SiC implanted with H_2_^+^ ions at 900 °C. This result demonstrates that the exfoliation efficiency of the 6H-SiC implanted with H_2_^+^ ions at 900 °C is higher than that of the 6H-SiC implanted with H_2_^+^ ions at 450 °C followed by 1100 °C annealing.

**Figure 4:**
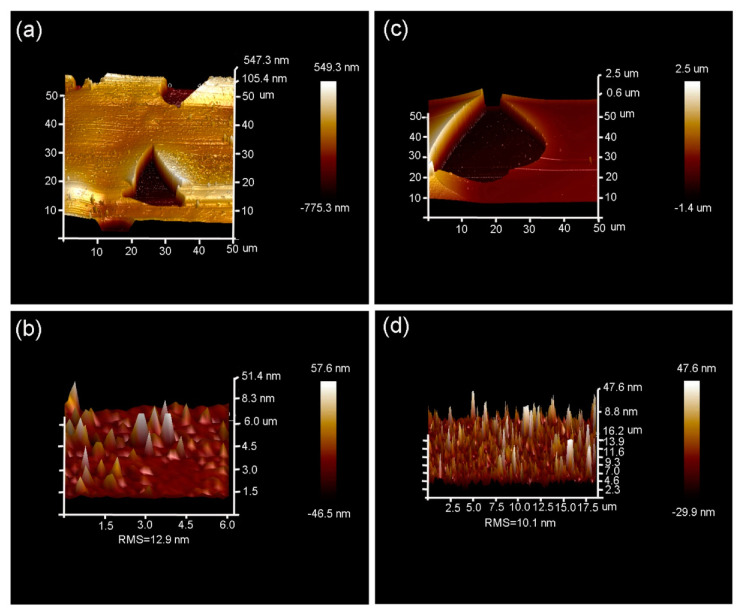
AFM images showing over-viewed surface morphology with a scanning zone of 50 × 50 μm^2^ of the 194 keV H_2_^+^-implanted 6H-SiC at 450 °C followed by 1100 °C annealing for 15 min (**a**), the 194 keV H_2_^+^-implanted 6H-SiC at 900 °C (**c**); (**b**,**d**) show the surface morphology of the exfoliation zone for the H_2_^+^-implanted 6H-SiC at 450 °C followed by 1100 °C annealing for 15 min and H_2_^+^-implanted 6H-SiC at 900 °C, respectively.

To carefully investigate the surface morphology after H_2_^+^-implantation into 6H-SiC, an AFM test was performed, and results are presented in Figure 4. It can be seen that the shape of the exfoliation zone is not regular, near an oval shape, as shown in Figure 4a, c. The formation of the exfoliation zone is due to the breakage of a surface blister when its inner stress exceeds the material fracture toughness [26,27,28]. The exfoliation zone is presented by a three-dimensional image, and the surface is not even, consisting of many hillocks. The values of the root-mean-square (RMS) roughness are 12.9 nm and 10.1 nm for the H_2_^+^-implanted 6H-SiC at 450 °C followed by 1100 °C annealing for 15 min and the H_2_^+^-implanted 6H-SiC at 900 °C, respectively. The decrease in RMS for the 6H-SiC implanted with H_2_^+^ ions at 900 °C is due to the fast growth of hydrogen-vacancy clusters, resulting in the increase in exfoliation efficiency, as observed by using the scanning white-light interferometry method (see Figure 3).

**Figure 5:**
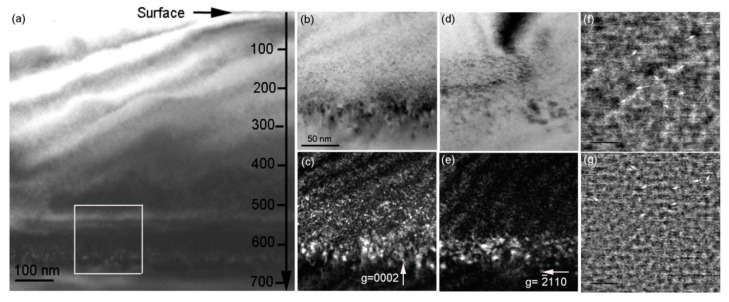
Bright-field XTEM image showing the over-viewed damage distribution in the 194 keV H_2_^+^-implanted 6H-SiC at 450 °C. The enlarged selected zone shown in (**a**) is presented in (**b**) to (**e**), where (**b**) BF and (**c**) WBDF images were observed under ***g*** = 0002 and (**d**,**e**) were observed under ***g*** = 2110. (**f**) The high-resolution transmission electron microscope image shows lattice defects exhibiting a dark contrast, as indicated by white arrows and (**g**) inverse Fourier-filtered image of (**f**) in order to improve the visibility of lattice defects. (**c**–**e**) have the same scale as (**b**).

To explain the higher exfoliation efficiency in the sample implanted at 900 °C compared to the sample implanted at 450 °C and consequently annealed at 1100 °C, microstructures of lattice defects and microcracks were investigated by XTEM. Figure 5 presents the general view of the lattice defects formed in the 194 keV H_2_^+^-implanted 6H-SiC at 450 °C. It can be seen in Figure 5a that the damage band exhibiting a black contrast is located at a depth ranging from approximately 540 to 650 nm beneath the surface. According to SRIM-2013 simulation, the projected range of 194 keV H_2_^+^ implantation is 553 nm with a straggling range of 52 nm. This implies that the measured damage band is deeper than the simulated projected range. This result can be accounted for by lattice swelling due to dense interstitial-type defects produced by H_2_^+^ implantation. It is reasonable to expect the lattice swelling to be approximately 5%. To investigate lattice defects in the damage band, bright-field (BF) and WBDF observations with two different diffraction vectors were performed, as shown in Figure 5b–e. Due to the nano-scaled lattice defects, these lattice defects are easily distinguished under the WBDF condition. It can be seen that many bright spots were observed, and some bright spots have larger sizes at the bottom of the damage band compared with the front of the damage band. The width of the observed damage band is approximately 120 nm. In the front of the damage band, many point defect clusters were observed under ***g*** = 0002, but not at ***g*** = 2110. This result indicated that these point defect clusters are Frank loops with a Burgers vector of 1/2<0001>. At the bottom of the damage band, some large defect clusters were observed under ***g*** = 0002 and ***g*** = 2110 simultaneously. It is indicated that these defect clusters have a Burgers vector of 1/6<2203>. The distribution of the lattice atoms was measured by high-resolution TEM (HRTEM), and the observed lattice defects exhibited a black contrast due to Bragg diffraction, as shown in Figure 5f. It can be seen in Figure 5f,g that lattice fringes are seriously disturbed by H_2_^+^ implantation. Because the C and Si vacancies cannot migrate at 450 °C, most of the observed lattice defects are composed of interstitial atoms, such as C interstitials, which induce a significant lattice swelling at 450 °C implantation [29,30].

**Figure 6:**
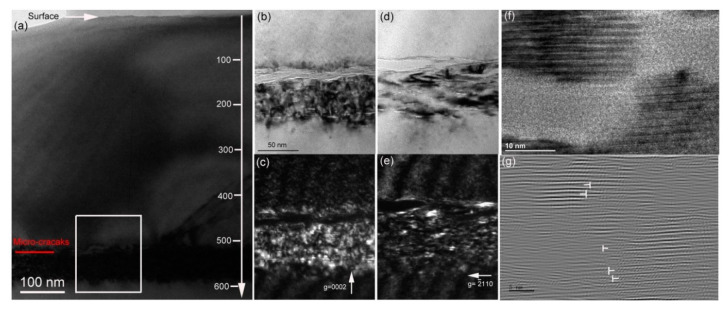
(**a**) Bright-field XTEM image showing the over-viewed damage distribution in the 194 keV H_2_^+^-implanted 6H-SiC at 450 °C followed by 1100 °C annealing for 15 min. The enlarged selected zone shown in (**a**) is presented in (**b**–**e**), where (**b**) BF and (**c**) WBDF images were observed under ***g*** = 0002, and (**d**,**e**) images were observed under ***g*** = 2110. (**f**) The high-resolution transmission electron microscope image shows the lattice fringe along the micro-crack, and (**g**) the inverse Fourier-filtered image of (**f**) shows interstitial-type dislocation loops on the (0001) plane. The scale in (**b**) is the same as that in (**c**–**e**).

Figure 6 presents the damage distribution in the 194 keV H_2_^+^-implanted 6H-SiC at 450 °C after 1100 °C annealing for 15 min. Compared with the microstructure observed in the as-implanted sample, after 1100 °C annealing, three evident changes can be observed. The first change is the depth distribution of the damage band. Figure 6a shows the over-viewed damage distribution where the damage band located at a depth ranging from 510 to 590 nm beneath the sample surface can be clearly distinguished. The observed damage band is shallower than that of the H_2_^+^-implanted 6H-SiC at 450 °C. This is attributed to defect recovery after 1100 °C annealing—Frank loops in particular. The observed damage band is well consistent with the simulated profiles. The second change is that a long microcrack parallel to the sample surface is observed in the front of the damage band. The formation of microcracks is due to the combined effects of Si vacancy migration at 1100 °C and the chemical interaction of H atoms and dangling bonds in the platelets [31,32]. The third change is that the width of the damage band observed under ***g*** = 0002 is the same as the case under ***g*** = 2110. The width of the damage band observed under ***g*** = 2110 increases after annealing. This is a reverse annealing phenomenon that is attributed to the growth of the microcrack accompanied by emitting interstitial atoms. A similar phenomenon was observed in He-implanted SiC [33,34]. The HRTEM image shown in Figure 6f confirms an amorphous structure inside the microcrack. Interstitial-type dislocation loops formed in the periphery of the microcrack are shown in Figure 6g. The microcrack is not straight, which induces the roughness of the exfoliation surface. The exfoliation surface was measured by AFM, and the result is shown in Figure 4.

**Figure 7:**
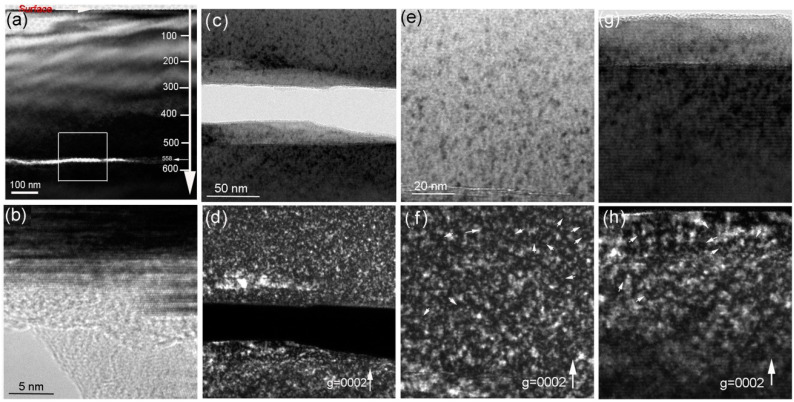
(**a**) Bright-field XTEM image showing the over-viewed damage distribution in the 194 keV H_2_^+^-implanted 6H-SiC at 900 °C. (**c**) BF image and (**d**) WBDF image under ***g*** = 0002 show the microstructure in the periphery of the microcrack. The magnified images taken from (**a**) show the lattice defects indicated by arrows above the microcrack presented in (**e**,**f**) and below the microcrack presented in (**g**,**h**). (**b**) HRTEM image shows the lattice fringe along the microcrack.

Figure 7 presents the damage distribution in the 194 keV H_2_^+^-implanted 6H-SiC at 900 °C. It can be seen in Figure 7a that a damage band is located at a depth ranging from 400 to 620 nm beneath the surface. Inside the damage band, a microcrack exhibiting bright contrast is located at 558 nm beneath the surface. Around the microcrack, dense Frank loops were observed, as shown in Figure 7d–g. The HRTEM image shows an amorphous structure inside the microcrack. Compared with the case of H_2_^+^-implanted 6H-SiC at 450 °C followed by 1100 °C annealing for 15 min, there are two significant differences in the 6H-SiC implanted with H_2_^+^ ions at 900 °C. The first difference is the location of the microcrack. The microcrack is in the front of the damage band of the 6H-SiC implanted with H_2_^+^ ions at 450 °C followed by 1100 °C annealing, whereas the microcrack is located at a depth between the damage peak and maximum hydrogen deposition simulated by SRIM-2013 for the 6H-SiC implanted with H_2_^+^ ions at 900 °C. The second difference is the width of the damage band. The width of the damage band increases significantly during implantation at 900 °C. Two reasons can account for this. One is the increasing vacancy-hydrogen interaction at 900 °C, but not at 450 °C [31]. The growth of vacancy-hydrogen clusters can emit interstitials and then these interstitials migrate and accumulate into Frank loops. The fast growth of the vacancy-hydrogen clusters leads to the occurrence of the microcrack, as observed in Figure 7a. The other is the influence of the sample surface, which acts as a defect sink. As shown in Figure 7a, some lattice defects were observed at a depth near 400 nm. It is indicated that some interstitials produced by H_2_^+^ collision migrate towards the sample surface during implantation at 900 °C. This is consistent with the defect distribution in the He-implanted SiC at elevated temperatures [35].

## 4. Discussion

Surface blisters and exfoliation of hydrogen implantation into 6H-SiC are attributed to the growth of microcracks inside the sample. When the amount of hydrogen is sufficient, this leads to internal pressure that is high enough to overcome the surface energy *γ*, and then to open up the crack. Matani and Gosele [36] have argued the critical radius for the on-set of blistering.

(1) $$r_{cric}={\left\{\frac{16\gamma Et^{3}}{9\alpha \left(1-v^{2}\right)\Delta p^{2}}\right\}}^{1/4}$$

where Δ*p* is the difference between the inside platelets and the outside atmosphere, *t* is the microcrack depth, *E* is the material’s Young’s modulus, *v* is Poisson’s ratio, α is a numerical factor in the order of ~1, and *γ* is the specific interface energy which would be changed by the implantation-induced defects. Freund [37] developed a model to explain wafer splitting via crack growth triggered by gas pressure *p*. The necessary condition for crack growth can be expressed as:

(2) $$p=\mu {\left(\frac{\pi }{1-v}\frac{\gamma }{a\mu }\right)}^{1/2}$$

where *µ* is the shear modulus, and *a* is the crack size. Based on Equation (2), the critical pressure required in the crack cavity decreases with the increase in the crack size.

The microstructure shows the size of the observed microcrack is larger in the 6H-SiC implanted with H_2_^+^ ions at 900 °C than that in the H_2_^+^-implanted 6H-SiC at 450 °C followed by 1100 °C annealing. This result indicates the fast growth of microcracks when the sample was implanted at a higher temperature, compared with the lower temperature implantation followed by a higher temperature annealing. According to Equation (2), the critical value of the inner pressure was easily achieved when the sample was implanted at 900 °C; therefore, the critical radius for the on-set of blistering increased when the sample was implanted at 900 °C based on Equation (1). Because the size of an exfoliation zone is smaller than its corresponding blister, the observed exfoliation size was far larger when the sample was implanted at 900 °C, as compared to the sample implanted at 450 °C and then annealed at 1100 °C.

Unlike C and Si interstitials, C and Si vacancies cannot migrate at 450 °C [31]. Dynamic annealing is, therefore, not significant and many survival defects are formed inside the sample. These defects lead to the evident lattice swelling. After annealing at 1100 °C for 15 min, an obvious microcrack was observed in the front of the damage band, where the lattice damage was smaller than the damage peak. It can be speculated that the specific interface energy *γ* increases with the increase in lattice defects [38]; therefore, the increase in *γ* needs more gas pressure of the crack in order to sustain crack growth. As a result, the crack growth is retarded in the peak damage region. When the sample was implanted at 900 °C, interstitials and vacancies can migrate simultaneously. The compound of hydrogen and vacancies can rapidly form and then coalesce into platelets. The growth of platelets is followed by pushing away of the matrix atoms. Named after the trap-mutation process [39,40], this can be expressed as:

H_n_V_m_→H_n_V_m+1_ + *I* (3)

Due to the rapid growth of platelets, dense self-interstitials were pushed away to form dislocation loops, which were observed by XTEM, as shown in Figure 7. This implies that the damage band is wider and the concentration of the observed lattice defects is higher in the sample implanted at 900 °C compared to the sample implanted at 450 °C. It should be noted that lattice swelling was significant when the sample was implanted at 450 °C, but not 900 °C. This is attributed to the limited resolution of conventional TEM (near 1 nm in WBDF), and hence there are many formed interstitials due to slow growth at 450 °C, which are too small to be observed by XTEM. After 1100 °C annealing, some interstitials migrated and recombined with vacancies to recover the lattice damage. Other interstitials migrated and coalesced into dislocation loops, which were observed under WBDF with ***g*** = 2110, as shown in Figure 6e. It should be discussed why the microcrack is located at a depth between the damage peak and the maximum hydrogen deposition for the sample implanted at 900 °C. Because of the rapid growth of vacancy-hydrogen clusters at 900 °C, available vacancies and hydrogen were higher in the middle of the two peaks than in other zones, and thus a microcrack easily grew in this zone, which was observed by XTEM (see Figure 7a).

The scanning white-light interferometry method showed that the exfoliation depth in the different exfoliation sites was almost the same. The formation of hillocks after exfoliation was due to the crack growth via the coalescence of platelets in the different depths; therefore, a zigzagged microcrack was observed by HRTEM, as shown in Figure 6f and Figure 7h. In the microcrack zone, an amorphous structure demonstrated the strong interaction between hydrogen and carbon/silicon dangling bonds on the inner surface of the microcrack. This is consistent with the report of Hojou et al. [41] that H_2_, C-H, and Si-H compounds were formed in the bubbles produced by H_2_^+^ + He^+^ simultaneously-implanted polycrystalline 6H-SiC. Therefore, it can be argued that hydrogen is effective in enhancing amorphization due to the chemical interaction. The surface morphology showed the exfoliation efficiency was higher for the sample implanted at 900 °C than that of the sample implanted 450 °C and subsequently annealed at 1100 °C. However, the formed damage band in the sample implanted at 900 °C was almost twice as high as that in the sample implanted at 450 °C and subsequently annealed at 1100 °C. The formed dislocation loops are stable and not easily annealed after thermal treatment [42,43,44]. To fabricate the SiCOI structure for the final electronic and optoelectronic device applications, these lattice damage zones after wafer transfer must be removed by the chemical mechanical polishing (CMP) process. To enable reuse of the wafer, the survival damage band should as narrow as possible. Therefore, low-temperature implantation, followed by high-temperature annealing is a better choice for the fabrication of the SiCOI structure compared with conventional implantation at a high temperature.

It should be noted that in nuclear fusion applications, dense energetic hydrogen can be produced in SiC by nuclear transmutations [45]. This hydrogen can interact with SiC forming displacement damage cascades and subsequently deposits in the near-surface layer. Because the first wall of the fusion reactor is expected to face a very high temperature, a comprehensive understanding of the surface exfoliation of SiC will require further study involving irradiation experiments at different temperatures and utilizing ion fluxes.

## 5. Conclusions

The exfoliation efficiency of H_2_^+^ implantation at 450 °C and 900 °C to a fluence of 5 × 10^16^ H_2_/cm^2^ in 6H-SiC was investigated. A lattice swelling of 5% was observed in the H_2_^+^-implanted 6H-SiC at 450 °C. Reverse dynamic annealing was observed in the H_2_^+^-implanted 6H-SiC at 900 °C. This is related to the rapid growth of hydrogen and vacancy clusters (H_n_V_m_), following by emitting interstitials around H_n_V_m_. A microcrack was observed in the front of the damage band in the sample implanted with H_2_^+^ ions at 450 °C and subsequently annealed at 1100 °C for 15 min. In the sample implanted with H_2_^+^ ions at 900 °C, a microcrack occurred between the displacement damage peak and the maximum hydrogen deposition. The change in specific interface energy *γ* can explain the location of the microcrack. Despite a high efficiency of exfoliation in the sample implanted at 900 °C, this procedure is not considered optimal for the fabrication of the SiCOI structure due to a wide damage band formed during H_2_^+^ implantation. Instead, we propose hydrogen implantation at a temperature lower than the critical temperature for vacancy migration as a more suitable method for this purpose.
